# Impact of Comorbid Generalized Anxiety Disorder on rTMS/iTBS Clinical Outcomes in Major Depression: A Multicenter Registry-Based Observational Study

**DOI:** 10.3390/jpm16020068

**Published:** 2026-01-30

**Authors:** Yoshihiro Noda, Ryota Osawa, Yuya Takeda, Keiko Fujita, Takumi Tsuji, Ryosuke Kitahata

**Affiliations:** 1 Department of Psychiatry, International University of Health and Welfare, Mita Hospital, Tokyo 108-8329, Japan; 2 Tokyo Yokohama TMS Clinic, Minato-Tokyo Branch, Tokyo 108-0023, Japan; 3 Tokyo Yokohama TMS Clinic, Kosugi-Kanagawa Branch, Kawasaki 211-0063, Japan; 4 Shinjuku-Yoyogi Mental Lab Clinic, Tokyo 151-0051, Japan

**Keywords:** major depressive disorder, generalized anxiety disorder, repetitive transcranial magnetic stimulation, intermittent theta-burst stimulation, registry study, benzodiazepines

## Abstract

**Background:** Major depressive disorder (MDD) is often accompanied by generalized anxiety disorder (GAD), a comorbidity linked to greater illness burden and potentially poorer outcomes. Repetitive transcranial magnetic stimulation (rTMS) and intermittent theta-burst stimulation (iTBS) are established treatments for MDD, yet the impact of comorbid GAD and concomitant medications remains unclear. This study aimed to compare rTMS/iTBS treatment outcomes between patients with MDD with and without comorbid GAD, and to examine the association between concomitant psychotropic medication use, stimulation protocol, and treatment response in a real-world clinical setting. **Methods:** We conducted a retrospective observational analysis using registry data from 108 patients (MDD + GAD: *n* = 36; MDD only: *n* = 72). Patients received either Left-iTBS or Right-rTMS. Baseline severity, percentage change in Montgomery–Åsberg Depression Rating Scale (MADRS) and Hamilton Depression Rating Scale (HAMD-17) scores, response, and remission were assessed. Logistic and linear regression models adjusted for age, sex, and baseline severity were applied. Sensitivity analyses stratified by stimulation protocol and benzodiazepine (BDZ) use were performed. **Results:** Baseline severity did not differ between groups. MADRS reduction was numerically lower in the comorbid GAD group (48.3% vs. 52.7%, *p* = 0.09), whereas HAMD-17 reduction was comparable. Response and remission rates did not differ significantly. Medication use and stimulation protocol did not show statistically significant independent associations with outcomes. Sensitivity analyses confirmed equivalent outcomes between Left-iTBS and Right-rTMS. BDZ users showed a non-significant trend toward lower MADRS improvement and remission. **Conclusions:** rTMS/iTBS produced substantial clinical improvement and was well tolerated in both patients with MDD and those with MDD comorbid with GAD. Although comorbid anxiety showed a modest tendency to attenuate MADRS score reduction, overall response and remission rates were comparable between groups. Neither concomitant medications nor stimulation protocol significantly affected treatment outcomes, while the potential influence of BDZ exposure warrants further investigation.

## 1. Introduction

Major depressive disorder (MDD) is a leading cause of disability worldwide and represents a heterogeneous clinical entity frequently complicated by psychiatric comorbidities [1,2,3]. Among these, generalized anxiety disorder (GAD) is particularly prevalent, with epidemiological studies reporting comorbidity rates exceeding 40% in clinical populations [4,5]. The coexistence of anxiety and depressive symptoms is associated with greater illness severity, poorer functional outcomes, and reduced responsiveness to conventional pharmacological and psychotherapeutic interventions [6,7,8,9]. From a personalized medicine perspective, comorbid GAD may represent a clinically relevant disease subtype within MDD, requiring tailored diagnostic and therapeutic strategies [4,7,8,9]. Understanding how comorbid GAD influences treatment response is therefore of considerable importance for optimizing patient selection, stratifying risk, and guiding individualized care [4,7,8,9].

Repetitive transcranial magnetic stimulation (rTMS) and intermittent theta-burst stimulation (iTBS) have emerged as evidence-based neuromodulatory treatments for MDD [10,11]. Both modalities modulate cortical excitability and neuroplasticity, with robust efficacy demonstrated in randomized controlled trials and meta-analyses [12,13]. However, the impact of comorbid anxiety disorders on rTMS/iTBS outcomes remains unclear. Some studies suggest that anxiety symptoms may attenuate antidepressant response, while others report comparable efficacy regardless of comorbidity [7,14,15]. Clarifying this relationship is essential not only for clinical practice but also for advancing precision psychiatry, where identifying subgroups likely to respond or fail to respond to neuromodulation is central to improving therapeutic outcomes [13,16,17].

Pharmacological factors may further influence neuromodulation efficacy [18]. Concomitant use of benzodiazepines has been hypothesized to reduce cortical excitability, potentially diminishing rTMS/iTBS effectiveness [19]. Similarly, antipsychotic or antidepressant co-medication may interact with neuromodulatory mechanisms in ways that alter treatment trajectories [20]. Yet, empirical evidence regarding these effects is inconsistent, and few studies have systematically examined medication use alongside stimulation protocols in the context of comorbid anxiety [21,22]. From a personalized medicine standpoint, these pharmacological moderators represent critical covariates that may help explain interindividual variability in treatment response and remission [17].

The present study aimed to compare rTMS/iTBS treatment outcomes between patients with MDD with and without comorbid GAD, thereby addressing whether comorbidity defines a distinct response phenotype. Specifically, we compared baseline severity, percentage reduction in depressive symptoms, response, and remission rates between the groups. We further examined the influence of concomitant medication use (antidepressants, antipsychotics, and benzodiazepines) and stimulation protocol (Left-iTBS vs. Right-rTMS) on treatment outcomes. Finally, sensitivity analyses were conducted to assess whether benzodiazepine exposure or stimulation protocol moderated treatment effects. By integrating clinical outcomes with pharmacological and procedural covariates, this study sought to elucidate the influence of comorbid anxiety and related factors on the efficacy of neuromodulation in MDD. Through this approach, it advances the broader objectives of personalized medicine: delineating diagnostic features of clinically relevant subtypes, identifying patient populations more or less likely to derive therapeutic benefit, and informing evidence-based strategies for individualized treatment planning [16,17].

## 2. Methods

### 2.1. Study Design and Participants

This study employed a retrospective observational design using TMS registry data [23] from patients diagnosed with major depressive disorder (MDD) who received rTMS therapy at three collaborative facilities (Tokyo Yokohama TMS Clinic, Minato-Tokyo Branch, Tokyo, Japan; Tokyo Yokohama TMS Clinic, Kosugi-Kanagawa Branch, Kanagawa, Japan; and Shinjuku-Yoyogi Mental Lab Clinic, Tokyo, Japan). Patients were categorized into two groups: those with comorbid generalized anxiety disorder (GAD) (*n* = 36) and those with MDD only (*n* = 72). The MDD-only group was randomly sampled from a larger cohort (*n* = 196) using a fixed random seed (set.seed(42)) prior to data sampling and statistical modeling, in order to ensure reproducibility and balance between groups. All random sampling and permutation procedures were conducted in R (version 4.3.1).

All diagnoses were established by board-certified psychiatrists according to the Diagnostic and Statistical Manual of Mental Disorders, Fifth Edition (DSM-5) criteria [24].

### 2.2. Treatment Protocols

Patients underwent either intermittent theta-burst stimulation (iTBS) applied to the left dorsolateral prefrontal cortex (Left-iTBS) or low-frequency 1 Hz repetitive transcranial magnetic stimulation (rTMS) applied to the right dorsolateral prefrontal cortex (Right-rTMS). In the majority of TMS treatments, the left dorsolateral prefrontal cortex (DLPFC) was designated as the stimulation target, and all participating clinics localized the site using the Beam F3 method [25]. When the right DLPFC was selected as the stimulation target, the corresponding location was determined by mirroring the Beam F3 coordinates across hemispheres. Stimulation parameters included the number of pulses (600, 1200, and 1800), stimulation intensity (100–120% of the resting motor threshold (RMT)), and the number of treatment sessions (typically 30). Treatment parameters were largely harmonized across participating sites; nevertheless, specific aspects, including stimulation protocol, intensity, number of pulses, session scheduling, and overall treatment duration, were individualized in a naturalistic fashion, guided by each patient’s clinical presentation, personal preferences, and the clinical judgment of the rTMS physician [26]. However, unless there were special circumstances on the part of the patient, the majority received TMS treatment three times per week and completed one treatment course over approximately ten weeks.

All participating clinics administered treatment using the MagPro R30 TMS system (MagVenture Inc., Farum, Denmark). Stimulation was delivered with Cool-B70 coils (MagVenture Inc., Farum, Denmark) in three clinics, while one clinic employed Cool-B65 coils (MagVenture Inc., Farum, Denmark) from the same manufacturer. Prior to commencing rTMS, each patient’s RMT was established, and stimulation intensity was uniformly set at 120% of RMT across sites. Consistent with common practice in TMS protocols for depression, RMT was determined by visual inspection: the threshold was defined as the lowest stimulus intensity at the left M1 hotspot that elicited visible muscle contractions in the right hand in at least 3 of 6 consecutive trials [27].

### 2.3. Concomitant Medications

Medication use was recorded at baseline, including antidepressants (AD), antipsychotics, and benzodiazepines (BDZ). These variables were coded dichotomously (present vs. absent) and included as covariates in subsequent analyses.

### 2.4. Outcome Measures

Depressive symptoms were assessed using the Montgomery–Åsberg Depression Rating Scale (MADRS) [28] and the 17-item Hamilton Depression Rating Scale (HAMD-17) [29]. Outcomes were assessed in the following sequence: baseline severity on the Montgomery–Åsberg Depression Rating Scale (MADRS baseline) and the 17-item Hamilton Depression Rating Scale (HAMD-17 baseline); percentage change in MADRS and HAMD-17 scores from baseline to post-treatment; treatment response defined as a ≥50% reduction from baseline on each scale; and remission defined as a post-treatment score ≤ 10 on MADRS and ≤7 on HAMD-17 [30]. Adverse events were recorded as binary outcomes (present vs. absent).

### 2.5. Statistical Analysis

**Figure 1:**
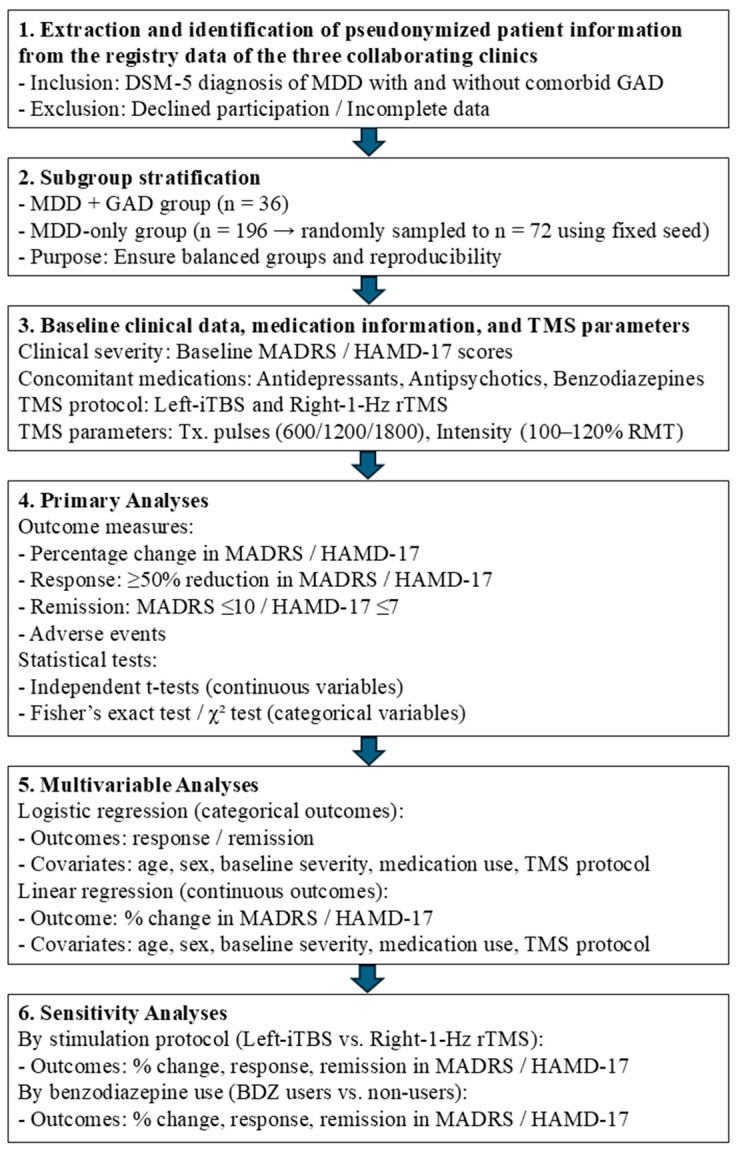
Study flow chart summarizing the analytic framework.

Figure 1 illustrates the study flowchart, which summarizes the analytic framework of the present research. Descriptive statistics were calculated for all variables. Continuous variables were expressed as mean ± standard deviation (SD) and compared between groups using independent samples *t*-tests. Categorical variables were expressed as percentages and compared using Fisher’s exact tests or chi-square tests, as appropriate.

Multivariable analyses were conducted to adjust for potential confounders. Logistic regression models were used for binary outcomes (response and remission), with age, sex, and baseline severity included as covariates. Results are reported as adjusted odds ratios (ORs) with 95% confidence intervals (CIs). Linear regression models were used for continuous outcomes (percentage change in MADRS and HAMD-17), with the same covariates included. Results are reported as regression coefficients (β) with 95% CIs.

Sensitivity analyses were conducted to evaluate the robustness of the findings, including the stability of estimates obtained from the logistic regression models. These analyses also allowed for a more direct assessment of whether specific attributes functioned as factors associated with treatment response by stratifying participants according to the presence or absence of each attribute and comparing treatment outcomes across the resulting subgroups. Accordingly, we stratified outcomes by stimulation protocol (left iTBS vs. right rTMS) and by BDZ use (present vs. absent) to determine whether treatment effects differed across subgroups.

All statistical analyses were conducted using IBM SPSS Statistics for Windows, version 29.0 (IBM Corp., Armonk, NY, USA). Continuous variables were summarized as means with standard deviations, whereas categorical variables were expressed as frequencies and percentages. A two-sided significance threshold of *p* < 0.05 was applied throughout.

Pseudonymized registry data from three clinics were used to identify patients with MDD, with or without comorbid GAD. After exclusions, participants were stratified into two balanced groups. Baseline data included symptom severity, psychotropic medication use, and TMS parameters. Primary and multivariable analyses assessed symptom change, response, remission, and adverse events. Sensitivity analyses examined the effects of stimulation protocol and benzodiazepine use.

### 2.6. Ethical Considerations

This investigation was conducted as an observational study utilizing registry data [23]. The study protocol received approval from the Research Ethics Committee of ITO Yoyogi Mental Clinic (Approval No. RKK319). This was implemented in collaboration with three partner clinics, which jointly agreed to extract and retrospectively analyze data from the TMS registry. Written informed consent was obtained from most participants prior to inclusion. For certain historical medical records in which obtaining individual consent was not reasonably feasible, an opt-out procedure approved by the Ethics Committee was implemented. Patients who declined participation were consequently excluded from the registry. All study procedures were conducted in accordance with the ethical principles outlined in the Declaration of Helsinki and complied with applicable national and institutional regulations governing research with human subjects.

## 3. Results

### 3.1. Patient Characteristics

A total of 108 patients were included in the analysis (MDD with comorbid GAD: *n* = 36; MDD only: *n* = 72, randomly sampled). The mean age was comparable between groups (36.2 ± 7.1 vs. 35.8 ± 6.9 years, *t* = −0.81, df = 106, *p* = 0.74). Sex distribution did not differ significantly (male proportion: 47.2% vs. 44.4%, Fisher’s exact test, *p* = 0.81). Concomitant medication use was similar across groups, including antipsychotics (11.1% vs. 8.3%, *p* = 0.72), BDZ (19.4% vs. 13.9%, *p* = 0.48), and antidepressants (16.7% vs. 15.3%, *p* = 0.88). The distribution of stimulation protocols was also balanced (Left-iTBS: 77.8% vs. 80.6%; Right-rTMS: 22.2% vs. 19.4%, χ^2^ = 0.11, df = 1, *p* = 0.74). Treatment parameters (number of pulses, stimulation intensity, number of sessions) and adverse event rates did not differ between groups.

### 3.2. Depressive Outcomes

Table 1 summarizes the primary outcomes in the prespecified order. Baseline severity was comparable between groups (MADRS-pre: 30.2 ± 5.1 vs. 31.0 ± 5.4, *p* = 0.42; HAMD-17-pre: 20.1 ± 3.8 vs. 21.0 ± 4.1, *p* = 0.29). The percentage reduction in MADRS scores tended to be lower in the comorbid GAD group (48.3 ± 16.2 vs. 52.7 ± 15.9, *t* = −1.70, df = 106, *p* = 0.09), whereas HAMD-17 reduction was similar (52.6 ± 14.8 vs. 54.1 ± 15.2, *p* = 0.61). Response and remission rates did not differ significantly for either scale (MADRS response: 66.7% vs. 72.2%, *p* = 0.53; HAMD-17 response: 72.2% vs. 75.0%, *p* = 0.78; MADRS remission: 38.9% vs. 43.1%, *p* = 0.68; HAMD-17 remission: 41.7% vs. 44.4%, *p* = 0.81).

### 3.3. Effect of Concomitant Medications

Across the entire sample, concomitant use of antipsychotics, BDZ, or antidepressants was not associated with significant differences in percentage change, response, or remission rates on either scale (See Table 2). Notably, BDZ non-users demonstrated a numerically greater reduction in MADRS scores (52.3 ± 15.9 vs. 47.5 ± 15.8), although this did not reach statistical significance (*t* = −1.31, df = 106, *p* = 0.19).

### 3.4. Effect of Stimulation Protocol

Patients treated with Left-iTBS and Right-rTMS exhibited comparable outcomes. Percentage change in MADRS (51.2 ± 15.8 vs. 52.0 ± 16.1, *p* = 0.76) and HAMD-17 (53.9 ± 15.0 vs. 54.5 ± 15.3, *p* = 0.82) did not differ (See Table 3). Response and remission rates were also equivalent (all *p* > 0.88).

### 3.5. Multivariable Analyses

Logistic regression models adjusted for age, sex, and baseline severity confirmed that neither concomitant medication use (antidepressant, antipsychotics, and BDZ) nor stimulation protocol independently predicted response or remission on MADRS or HAMD-17 (all adjusted OR ≈ 1.0, *p* > 0.56) (See Table 4). Linear regression models for percentage change outcomes similarly revealed no significant associations. BDZ use showed a small negative trend for MADRS reduction (β = −3.2, 95% CI −6.8 to 0.4, *t* = −1.74, *df* = 106, *p* = 0.08), but this did not reach conventional significance (See Table 5).

### 3.6. Sensitivity Analyses

#### 3.6.1. Stimulation Protocol

Sensitivity analyses stratified by stimulation protocol confirmed the absence of differential effects. Both Left-iTBS and Right-rTMS yielded comparable reductions in MADRS and HAMD-17 scores, with similar response and remission rates (all *p* > 0.76) (See Table 6).

#### 3.6.2. BDZ Use

Stratification by BDZ use suggested that non-users experienced slightly greater improvement in MADRS scores (52.3 ± 15.9 vs. 47.5 ± 15.8) and higher remission rates (42.7% vs. 34.3%). However, none of these differences achieved statistical significance (all *p* > 0.19) (See Table 7).

#### 3.6.3. Stimulation Parameters

Analyses of stimulation parameters, including total number of pulses (600, 1200, or 1800) and stimulation intensity (100–120% of resting motor threshold), revealed no significant associations with clinical outcomes in this cohort. Neither percentage changes in MADRS/HAMD-17 scores nor response/remission rates differed across parameter settings (all *p* > 0.10).

### 3.7. Adverse Events

Adverse events were limited to transient scalp discomfort and pain at the stimulation site. No serious adverse events, such as manic switch or seizure induction, were observed across the sample.

## 4. Discussion

### 4.1. Summary of Findings

In this study, we compared the clinical outcomes of rTMS and iTBS in patients with MDD with and without comorbid GAD. Across multiple outcome domains, including baseline severity, percentage reduction in depressive symptoms, response, and remission rates, patients with comorbid GAD demonstrated broadly comparable treatment effects to those with MDD alone. While the MADRS percentage reduction was numerically lower in the comorbid group, this difference did not reach statistical significance. To avoid overstating the implications of these findings, we interpret our results as indicating an absence of evidence for reduced rTMS/iTBS effectiveness in the presence of comorbid GAD, rather than evidence of equivalent efficacy across groups. This distinction is important given the methodological and statistical constraints of the present study.

### 4.2. Clinical Implications of Comorbidity

The observation of a non-significant trend toward reduced MADRS improvement in the comorbid group is consistent with prior literature indicating that anxiety symptoms can complicate the trajectory of depressive treatment response [6,31]. Although our findings do not establish a robust inhibitory effect, they highlight the need for clinicians to monitor comorbid anxiety carefully, as it may contribute to residual symptoms [32,33] or slower recovery [34,35]. Future trials with larger sample sizes may clarify whether this trend represents a clinically meaningful difference. Importantly, this trend did not reach statistical significance. Given the limited sample size of the comorbid GAD subgroup, the study was not sufficiently powered to confirm or refute such small effect differences. We therefore interpret these numerical differences cautiously and avoid overstating their clinical relevance.

### 4.3. Role of Concomitant Medications

Neither antidepressant, antipsychotic, nor BDZ use independently predicted treatment outcomes in adjusted models. This suggests that rTMS/iTBS efficacy is largely preserved regardless of concomitant pharmacotherapy [20,26]. Nevertheless, sensitivity analyses revealed a small, non-significant trend toward reduced MADRS improvement among BDZ users, as shown in Table 6 and Table 7, consistent with mechanistic concerns that BDZ may dampen cortical excitability and interfere with neuroplasticity [18,36,37,38,39]. Although this trend did not reach statistical significance, it should be interpreted in the context of limited statistical power. In this sample, only moderate-to-large effects were likely to be detectable, and smaller effects may have gone undetected.

### 4.4. Effects of TMS Protocols and Parameters

No differences were observed between Left-iTBS and Right-rTMS in terms of percentage reduction, response, or remission rates. These results support the clinical interchangeability of the two protocols in the context of MDD, regardless of comorbid anxiety. This finding is reassuring for practitioners, as it suggests flexibility in protocol selection without compromising efficacy [40,41,42]. Our analyses demonstrated that variation in stimulation parameters (pulse number and intensity) did not significantly affect treatment outcomes. This finding suggests that within the commonly applied clinical range, rTMS/iTBS efficacy is robust to modest differences in dosing parameters [43,44], supporting the generalizability of registry-based protocols.

### 4.5. Adverse Events in Comorbid Anxiety Disorders

Consistent with prior safety literature, adverse events in this cohort were restricted to mild scalp pain at the stimulation site. Importantly, no severe adverse events, including manic switch or seizure induction, were reported. These results reinforce the favorable tolerability profile of rTMS/iTBS in routine clinical practice [13,26,45], even among patients with comorbid anxiety.

### 4.6. Strengths of This Study

Strengths of this study include the utilization of real-world TMS registry data [46,47], the extraction of a clinically well-defined sample [20], the application of random sampling within the MDD-only group to balance group sizes, and the comprehensive adjustment for age, sex, and baseline severity in multivariable models [48,49].

### 4.7. Limitations

First, the modest overall sample size, particularly the small comorbid GAD subgroup, may have reduced statistical power to detect small but clinically meaningful differences. The study was powered to detect only moderate effects, and smaller differences in response or remission may therefore have gone undetected, raising the possibility of Type II error, particularly for subgroup and medication analyses. Non-significant trends, especially those related to anxiety comorbidity and BDZ use, should be interpreted cautiously and regarded as exploratory rather than confirmatory.

Second, anxiety was operationalized as a binary DSM-5 diagnosis. This categorical approach does not capture anxiety severity, symptom dimensions, chronicity, or overlap with depressive features. Therefore, the approach may lack the sensitivity needed to detect more nuanced moderators of neuromodulation responses.

Third, the handling of concomitant medication exposure entails additional constraints. Antidepressants, antipsychotics, and benzodiazepines were coded as simple present/absent variables, which do not account for dose, duration, timing relative to stimulation, or cumulative sedative burden. This limitation is particularly relevant for benzodiazepines, given mechanistic and empirical evidence suggesting dose-dependent effects on cortical excitability. Accordingly, our conclusion that medications “did not show statistically significant independent associations” should be interpreted with caution, as our measurement approach may not have been sensitive enough to detect more subtle or dose-related influences.

Finally, several analytical and design-related limitations should be noted. The reliance on percentage-change outcomes assumes linear symptom trajectories and may obscure clinically meaningful early, delayed, or non-linear response patterns [50,51]. Although baseline severity was comparable between groups, this analytic choice limits interpretability in a heterogeneous real-world cohort. Moreover, the observational design precludes causal inference [52,53,54], and despite random sampling to improve group balance, residual confounding related to illness chronicity, treatment resistance, or psychosocial factors cannot be fully excluded.

### 4.8. Future Directions

Future research should incorporate dimensional assessments of anxiety, including severity, symptom clusters, and chronicity, to better characterize how anxiety influences neuromodulation outcomes. Studies with larger samples will be essential to clarify whether the trends observed here represent true clinical differences. Additionally, more granular measurement of medication exposure, including dose, duration, and timing, will be necessary to elucidate potential dose-dependent interactions with cortical excitability.

At this stage, it will be important to examine whether specific patient subgroups, such as individuals with high baseline anxiety severity, those with prolonged BDZ exposure, or those exhibiting distinct clinical phenotypes, demonstrate differential responses to neuromodulation [13,55]. From a personalized medicine perspective, such subgroup analyses are critical for delineating diagnostic characteristics that define clinically meaningful subtypes of MDD [16]. However, while the conceptual rationale for examining comorbidity and medication effects aligns with personalized treatment approaches, the present analyses remain limited to subgroup comparisons and do not yet operationalize individualized prediction or stratification. Advancing toward true personalization will require future studies that incorporate dimensional symptom measures, mechanistic biomarkers, and predictive modeling frameworks capable of identifying individualized response profiles.

Incorporating neurophysiological markers of cortical excitability, such as motor threshold variability, EEG-based indices of network activity, or neuroimaging correlates of prefrontal function, may help elucidate mechanistic pathways underlying the observed trends [56,57]. These biomarkers could serve as objective tools to refine patient selection, monitor treatment progress, and predict outcomes, thereby bridging the gap between mechanistic neuroscience and individualized clinical care [16]. Moreover, integrating biological markers with clinical covariates such as comorbid anxiety or pharmacological exposure aligns with the broader goals of precision psychiatry [58,59,60], which seeks to move beyond “one-size-fits-all” approaches toward evidence-based personalization [61,62].

Randomized controlled trials with larger samples and stratified analyses are warranted to confirm whether comorbid anxiety modestly attenuates rTMS/iTBS efficacy [13,55]. Such trials should incorporate adaptive designs that allow subgroup-specific hypotheses to be tested prospectively [50]. In addition, longitudinal studies examining sustained outcomes and relapse prevention in patients with and without comorbid GAD would provide valuable insights into the durability of neuromodulation effects [63,64]. Importantly, future research should also consider regulatory, ethical, and policy dimensions, for example, how stratified treatment recommendations might influence access to neuromodulation, reimbursement frameworks, and equity in mental health care delivery [65,66].

Taken together, these directions highlight the need for a multidimensional research agenda that integrates clinical, neurophysiological, pharmacological, and socio-ethical perspectives [66,67]. By advancing knowledge of how comorbid anxiety and related covariates shape neuromodulation outcomes, future studies can contribute to the overarching mission of personalized medicine: to identify patient subgroups most likely to respond, to minimize ineffective treatment exposure, and to optimize individualized therapeutic strategies for major depressive disorder [63,64].

## 5. Conclusions

Our findings indicate that rTMS/iTBS is effective and well tolerated in patients with MDD, and provide no statistically significant evidence that comorbid GAD reduces treatment effectiveness in this real-world sample. While comorbid anxiety was associated with a non-significant trend toward smaller improvement in depressive symptoms, overall response and remission rates were comparable [68,69]. Concomitant medication use and stimulation protocol were not associated with significant independent effects in this sample [36,70]. These results support the clinical utility of rTMS/iTBS across heterogeneous patient populations, while highlighting the need for further investigation into the nuanced role of comorbid anxiety and BDZ exposure [36].

## Figures and Tables

**Table 1:** Comparison of depressive outcomes between patients with MDD and those with comorbid GAD.

Outcome	MDD with GAD (*n* = 36)	MDD Only (*n* = 72)	Statistic (*t*/*χ*^2^, *df*)	*p* Value
MADRS baseline	30.2 ± 5.1	31.0 ± 5.4	*t* = −0.81, *df* = 106	0.42
HAMD-17 baseline	20.1 ± 3.8	21.0 ± 4.1	*t* = −1.06, *df* = 106	0.29
% change in MADRS score	48.3 ± 16.2	52.7 ± 15.9	*t* = −1.70, *df* = 106	0.09
% change in HAMD-17 score	52.6 ± 14.8	54.1 ± 15.2	*t* = −0.51, *df* = 106	0.61
MADRS response	66.7%	72.2%	*χ*^2^ = 0.40, *df* = 1	0.53
HAMD-17 response	72.2%	75.0%	*χ*^2^ = 0.08, *df* = 1	0.78
MADRS remission	38.9%	43.1%	*χ*^2^ = 0.17, *df* = 1	0.68
HAMD-17 remission	41.7%	44.4%	*χ*^2^ = 0.06, *df* = 1	0.81

Footnotes: Values are presented as mean ± standard deviation (SD) for continuous variables and percentages for categorical variables. Response was defined as ≥50% reduction from baseline; remission was defined as MADRS ≤ 10 or HAMD-17 ≤ 7. Independent samples *t*-tests were used for continuous variables; Fisher’s exact tests for categorical variables.

**Table 2:** Depressive outcomes stratified by concomitant medication use across the full sample.

Medication	Outcome	On-Medication Group	No Medication Group	Statistic (*t*, *df*)	*p* Value
Antidepressants	% change in MADRS score	48.9 ± 16.1	52.0 ± 15.8	*t* = −1.00, *df* = 106	0.32
	% change in HAMD-17 score	52.8 ± 14.7	54.2 ± 15.1	*t* = −0.48, *df* = 106	0.63
Antipsychotics	% change in MADRS score	49.1 ± 16.0	51.8 ± 15.7	*t* = −0.73, *df* = 106	0.47
	% change in HAMD-17 score	53.2 ± 15.1	54.0 ± 15.0	*t* = −0.20, *df* = 106	0.84
BDZ	% change in MADRS score	47.5 ± 15.8	52.3 ± 15.9	*t* = −1.31, *df* = 106	0.19
	% change in HAMD-17 score	51.0 ± 14.9	54.8 ± 15.2	*t* = −1.09, *df* = 106	0.28

Footnotes: Outcomes represent percentage change from baseline to post-treatment. Comparisons were conducted using independent samples *t*-tests.

**Table 3:** Depressive outcomes according to stimulation protocol (Left-iTBS vs. Right-rTMS).

Outcome	Left-iTBS	Right-rTMS	Statistic (*t*/*χ*^2^, *df*)	*p* Value
% change in MADRS score	51.2 ± 15.8	52.0 ± 16.1	*t* = −0.30, *df* = 106	0.76
% change in HAMD-17 score	53.9 ± 15.0	54.5 ± 15.3	*t* = −0.23, *df* = 106	0.82
MADRS response	71.0%	70.0%	*χ*^2^ = 0.02, *df* = 1	0.88
HAMD-17 response	74.1%	73.3%	*χ*^2^ = 0.01, *df* = 1	0.91
MADRS remission	40.0%	39.6%	*χ*^2^ = 0.01, *df* = 1	0.93
HAMD-17 remission	42.0%	41.7%	*χ*^2^ = 0.00, *df* = 1	0.95

Footnotes: Left-iTBS = intermittent theta-burst stimulation applied to the left dorsolateral prefrontal cortex. Right-rTMS = 1 Hz repetitive transcranial magnetic stimulation applied to the right dorsolateral prefrontal cortex. Response and remission definitions follow those in Table 1. Fisher’s exact tests were used for categorical outcomes; *t*-tests for continuous outcomes.

**Table 4:** Logistic regression analyses of response and remission, adjusted for age, sex, and baseline severity as covariates.

Outcome	Independent Variable	Adjusted OR	95% CI	Wald *χ*^2^ (*df* = 1)	*p* Value
MADRS response	BDZ	0.90	0.56–1.46	*χ*^2^ = 0.17	0.68
	Antipsychotics	1.01	0.57–1.81	*χ*^2^ = 0.01	0.97
	Antidepressants	1.08	0.67–1.74	*χ*^2^ = 0.09	0.76
	TMS protocol	1.05	0.67–1.64	*χ*^2^ = 0.04	0.84
HAMD-17 response	BDZ	0.92	0.57–1.49	*χ*^2^ = 0.12	0.73
	Antipsychotics	0.98	0.55–1.75	*χ*^2^ = 0.01	0.94
	Antidepressants	1.07	0.66–1.73	*χ*^2^ = 0.07	0.78
	TMS protocol	1.03	0.66–1.61	*χ*^2^ = 0.02	0.89
MADRS remission	BDZ	0.88	0.54–1.45	*χ*^2^ = 0.26	0.61
	Antipsychotics	0.97	0.51–1.85	*χ*^2^ = 0.01	0.93
	Antidepressants	1.09	0.67–1.76	*χ*^2^ = 0.10	0.75
	TMS protocol	1.04	0.66–1.66	*χ*^2^ = 0.03	0.87
HAMD-17 remission	BDZ	0.86	0.51–1.44	*χ*^2^ = 0.34	0.56
	Antipsychotics	0.95	0.49–1.86	*χ*^2^ = 0.02	0.88
	Antidepressants	1.10	0.67–1.81	*χ*^2^ = 0.13	0.72
	TMS protocol	1.02	0.63–1.66	*χ*^2^ = 0.01	0.93

Footnotes: OR = odds ratio; CI = confidence interval. BDZ = benzodiazepines. Models adjusted for age, sex, and baseline MADRS/HAMD-17 scores. Wald χ^2^ statistics are reported with df = 1. Response = ≥50% reduction from baseline; remission = MADRS ≤ 10 or HAMD-17 ≤ 7.

**Table 5:** Linear regression analyses of percentage change in MADRS scores, adjusted for age, sex, and baseline severity as covariates.

Outcome	Independent Variable	β	95% CI	Statistic (*t*, *df*)	*p* Value
% change in MADRS score	BDZ	−3.2	−6.8 to 0.4	*t* = −1.74, *df* = 106	**0.08**
	Antipsychotics	−1.8	−5.6 to 2.0	*t* = −0.93, *df* = 106	0.35
	Antidepressants	−2.4	−5.9 to 1.1	*t* = −1.35, *df* = 106	0.18

Footnotes: β = regression coefficient; CI = confidence interval. BDZ = benzodiazepines. Models adjusted for age, sex, and baseline severity. Dependent variable = percentage change in MADRS scores. A negative β indicates a smaller improvement relative to the reference group. The boldface in the table indicates a trend toward statistical significance.

**Table 6:** Sensitivity analysis of depressive outcomes by stimulation protocol (Left-iTBS vs. Right-rTMS).

Outcome	Left-iTBS (*n* = 86)	Right-rTMS (*n* = 22)	Statistic (*t*/*χ*^2^, *df*)	*p* Value
MADRS baseline	30.8 ± 5.3	30.5 ± 5.2	*t* = 0.23, *df* = 106	0.82
HAMD-17 baseline	20.7 ± 4.0	20.5 ± 3.9	*t* = 0.19, *df* = 106	0.85
% change in MADRS score	51.2 ± 15.8	52.0 ± 16.1	*t* = −0.30, *df* = 106	0.76
% change in HAMD-17 score	53.9 ± 15.0	54.5 ± 15.3	*t* = −0.23, *df* = 106	0.82
MADRS response	71.0%	70.0%	*χ*^2^ = 0.02, *df* = 1	0.88
HAMD-17 response	74.1%	73.3%	*χ*^2^ = 0.01, *df* = 1	0.91
MADRS remission	40.0%	39.6%	*χ*^2^ = 0.01, *df* = 1	0.93
HAMD-17 remission	42.0%	41.7%	*χ*^2^ = 0.00, *df* = 1	0.95

Footnotes: Left-iTBS vs. Right-rTMS subgroup comparison. Outcomes are defined as in Table 1. No significant differences were observed across protocols.

**Table 7:** Sensitivity analysis of depressive outcomes by BDZ use.

Outcome	BDZ Users (*n* = 17)	BDZ Non-Users (*n* = 91)	Statistic (*t*/*χ*^2^, *df*)	*p* Value
MADRS baseline	30.5 ± 5.0	30.9 ± 5.3	*t* = −0.32, *df* = 106	0.75
HAMD-17 baseline	20.6 ± 3.9	20.8 ± 4.0	*t* = −0.18, *df* = 106	0.86
% change in MADRS score	47.5 ± 15.8	52.3 ± 15.9	*t* = −1.31, *df* = 106	0.19
% change in HAMD-17 score	51.0 ± 14.9	54.8 ± 15.2	*t* = −1.09, *df* = 106	0.28
MADRS response	68.6%	72.0%	*χ*^2^ = 0.16, *df* = 1	0.69
HAMD-17 response	71.4%	74.2%	*χ*^2^ = 0.09, *df* = 1	0.77
MADRS remission	34.3%	42.7%	*χ*^2^ = 0.77, *df* = 1	0.38
HAMD-17 remission	35.7%	44.0%	*χ*^2^ = 0.68, *df* = 1	0.41

Footnotes: BDZ = benzodiazepine. Outcomes are defined as in Table 1. Non-users demonstrated numerically greater improvement, but differences did not reach statistical significance.

## Data Availability

The data presented in this study are available on reasonable request from the corresponding author. Access is restricted in accordance with contractual agreements governing the use of the TMS registry. Data may be obtained either through the establishment of a formal data-use agreement or by institutional membership in the international TMS registry project, facilitated via the corresponding author.

## Abbreviations

The following abbreviations are used in this manuscript:

AD	Antidepressants
BDZ	Benzodiazepines
CI	Confidence Interval
DLPFC	dorsolateral prefrontal cortex
DSM-5	Diagnostic and Statistical Manual of Mental Disorders, Fifth Edition
HAMD-17	Hamilton Depression Rating Scale, 17-item version
iTBS	Intermittent Theta-Burst Stimulation
MADRS	Montgomery–Åsberg Depression Rating Scale
MDD	Major Depressive Disorder
OR	Odds Ratio
rTMS	Repetitive Transcranial Magnetic Stimulation
RMT	Resting Motor Threshold
SD	Standard Deviation
